# Caught in a trap: How pre-clinical studies in laboratory mice exaggerate vaccine responses

**DOI:** 10.1016/j.xcrm.2021.100484

**Published:** 2021-12-21

**Authors:** Lynda Coughlan

**Affiliations:** 1 Department of Microbiology and Immunology, Center for Vaccine Development and Global Health, University of Maryland School of Medicine, Baltimore, MD 21201, USA

## Abstract

In a recent issue of *Cell Host and Microbe*, Fiege and colleagues^1^ report that laboratory mice exposed to pathogens from pet-store mice exhibit impaired humoral immunity to influenza vaccination and display gene expression signatures that more authentically reflect human vaccine responses.

## Main text

The use of laboratory mice to evaluate infection and to measure the immunogenicity, efficacy, or mode of action of vaccines has been a crucial tool in medical research. Studies in mice have advanced our understanding of how specific genes contribute to disease and have enabled the identification of effectors involved in antigen presentation and the development of protective immunity. This fundamental knowledge has underpinned the development of vaccines, therapeutics, and licensed drugs.

Specific-pathogen-free (SPF) mice are free from a defined list of viruses, bacteria, or parasites. These animals are housed in clean conditions that minimize exposure to pathogens which could introduce uncontrolled variables into experiments. There are many advantages to using laboratory mice: they are small and easy to house, extensive reagents are available, diverse genetic backgrounds exist, and we have the capability to engineer genetically modified transgenic strains and humanized mice or produce bone marrow chimeric mice. Collectively, these factors allow rigorous, detailed investigations into mechanisms underlying disease, correlates of protection, or responsiveness to vaccination.

Despite the utility of mice in pre-clinical studies, they often fail to recapitulate hallmarks of human disease, and findings in mice do not always translate into humans. Diverse factors contribute to this, including genetic and physiological differences, species-specific characteristics of pathogens, or sex differences. Recently, consideration has been given to the role of environmental factors in modulating host immune outcomes following infection and/or immunization, including diet/nutritional status,^2^^,^^3^ microbiome,^4^ baseline inflammatory state,^5^^,^^6^ as well as the impact of co-infection or prior pathogen exposure, on innate or adaptive immunity.^1^^,^^7^

**Figure 1:**
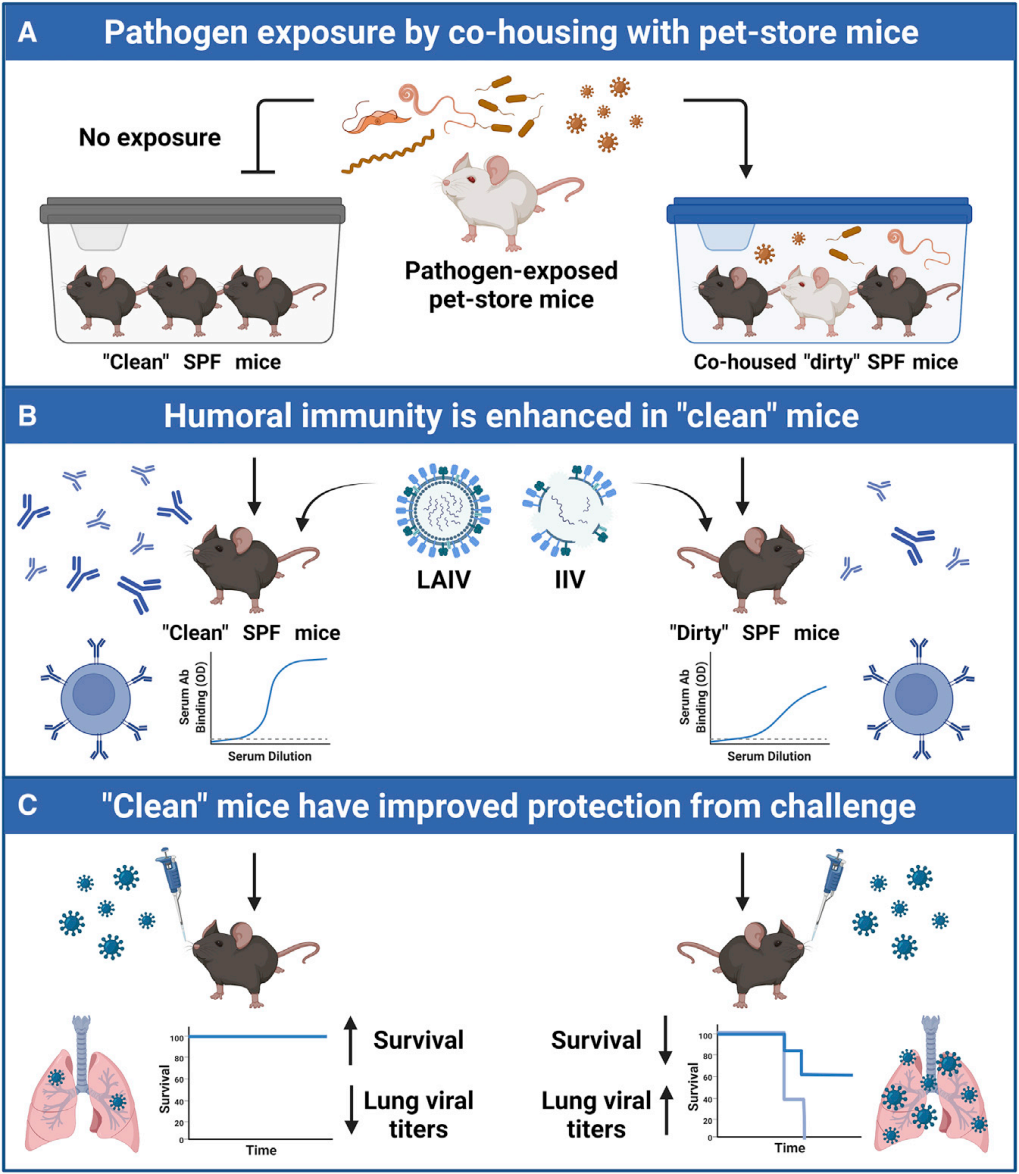
Pathogen-exposure affects vaccine immunogenicity and efficacy in mice (A) SPF “clean” mice or “dirty” SPF mice exposed to murine pathogens following co-housing with pet-store mice. (B) Immunization of clean mice resulted in higher titers of neutralizing antibodies and IgG subclass antibodies than dirty mice. (C) Improved humoral immunogenicity results in increased protection from influenza challenge than dirty mice. SPF, specific-pathogen free; LAIV, live attenuated influenza vaccine; IIV, inactivated influenza vaccine; OD, optical density. Created with BioRender.com.

There is growing evidence that exposure to pathogens can mature the murine immune system, resulting in an antigen-experienced state that may more accurately reflect immunological maturity in humans.^8^^,^^9^ To stimulate a pathogen-exposed basal state in mice, Fiege et al.^1^ co-housed SPF mice with pet-store mice to facilitate their exposure to diverse microbes (Figure 1A). Increased frequencies of activated CD8^+^ T cells were observed in co-housed “dirty” mice.^9^ The authors performed a series of experiments to assess how such differences might affect subsequent influenza virus infection or immunization with influenza vaccines (Figures 1B and 1C).

Gene expression profiles in cells from dirty or SPF mice prior to and 3 days post-immunization with an inactivated influenza vaccine (IIV) were measured. Drawing from existing transcriptomic datasets from humans immunized with similar IIVs at the same time-points, the authors compared post-immunization gene signatures in both species. They determined that vaccine-responsive genes in dirty mice more accurately mirrored responses in humans. Therefore, pre-clinical vaccine studies performed in dirty mice might be a more relevant predictor for vaccine responses in humans. To interrogate this in more detail, mice were challenged with influenza H1N1 strain A/Puerto Rico/8/1934 (PR8) or pandemic H1N1 strain A/California/04/2009. Primary responses to infection were similar, suggesting that immune-experience did not appear to affect the pathogenesis of wildtype influenza infection. However, when the authors immunized dirty and SPF mice with live attenuated influenza vaccine (LAIV), split-virion IIV, and adjuvanted IIV platforms, they found that dirty mice displayed impaired induction of humoral immunity with reduced titers of neutralizing antibodies (NAbs), reduced binding of virus-specific IgG subclasses, and a limited capacity to control viral replication in the lung.

The development of universal influenza virus vaccines that elicit cellular immunity against conserved internal antigens such as nucleoprotein (NP) is underway.^10^ Therefore, the authors assessed the impact of exposure to H3N2 (X31) on subsequent challenge with PR8 in SPF or dirty mice. In this model, the internal antigens of both viruses are matched but the hemagglutinin (HA) and neuraminidase (NA) glycoproteins are mismatched, allowing an evaluation of heterosubtypic protection in the absence of strain-specific antibodies. SPF mice had robust protection from morbidity (limited weight loss, reduced viral lung titers) and complete protection from mortality. In contrast, dirty mice were not protected. Although no distinct differences in T cell function or frequency were observed, increases in antigen-specific CD8^+^ T cells were detected in the lung parenchyma of dirty mice prior to challenge. The implications of this were not defined, but will undoubtedly be the subject of future work. In conclusion, the authors proposed that SPF mice mount exaggerated responses to immunization that result in superior protection from challenge. Considering that the majority of next-generation influenza vaccines are initially evaluated in SPF mice, exclusive use of these mice may limit subsequent translational relevance, particularly in light of the authors’ findings that vaccine responsive gene expression in dirty mice mimics adult humans.

The factors that affect the efficacy of influenza vaccines are complex, and a single mouse model cannot address all hypotheses. Immunogenicity or efficacy depends on the antigen or epitope target, the vaccine platform or formulation, the route of administration, the phenotype of immunity, as well as prior exposure to influenza viruses. Indeed, the extensive history of exposure to influenza viruses in humans is a major confounding factor when translating pre-clinical studies into humans. Nonetheless, the findings described by Fiege and colleagues^1^ are intriguing. Further characterization of the precise differences between SPF and dirty mice, namely, potential defects in germinal center (GC) formation/function that result in impaired Ab responses in dirty mice or alternative pathways relevant to protection from influenza virus, are warranted. These data may shed light on new targets for rationally designed adjuvants or vaccine formulations or could help identify strategies to modulate the basal immune state to enhance immunogenicity.^5^ Pre-vaccination immune signatures associated with differential outcomes in humoral immunity have been identified in humans.^6^ Gaining a more comprehensive understanding of how basal immune activation signatures affect vaccine efficacy and how environment and pathogen-exposure contribute to this may enable us to harness pathways to design improved next-generation vaccines or immunization regimens.
